# Colorectal Cancer Presenting as Single Pulmonary Hilar Lymph Node Metastasis

**DOI:** 10.1155/2018/5474919

**Published:** 2018-01-18

**Authors:** Ahmed M. Habib, Xenophon Kassianides, Samuel Chan, Mahmoud Loubani, Syed Qadri

**Affiliations:** ^1^Cardiothoracic Surgery Department, Castle Hill Hospital, Cottingham, UK; ^2^Cardiothoracic Surgery, Ain Shams University Hospitals, Cairo, Egypt; ^3^Respiratory Department, York District Hospital, York, UK

## Abstract

Colorectal carcinoma is the second biggest cancer responsible for mortality. Lung metastasis is the commonest, following the liver. It is not uncommon to perform pulmonary metastasectomy and identify mediastinal metastasis. Previous studies have identified incidental lymph node involvement following routine mediastinal lymph node clearance in 20–50% of cases. However, solitary intrathoracic lymph node metastasis is exceedingly rare. Even when present, it is usually metachronous. In our case, we present an exceedingly rare case whereby the intrathoracic lymph node metastasis is solitary, not accompanying pulmonary disease and with no liver metastasis. We also review the evidence for mediastinal lymphadenectomy in the literature.

## 1. Introduction

Mortality from colorectal cancer is the second only to lung cancer [1], with 19% of the cases having systemic disease at presentation [2]. Its management and route of metastasis have been extensively investigated, with hepatic metastases being the most frequent; extrahepatic spread includes the perihepatic lymph nodes, pulmonary and mediastinal lymph nodes, peritoneum, bone, and brain [3]. Routine mediastinal lymph node clearance during pulmonary metastasectomy identified that 20–50% of cases have nodal involvement [4]. It is extremely rare for colorectal cancer to present with single pulmonary lymph node involvement in the absence of metastasis elsewhere. Paravertebral venous plexus could be a route for colorectal cancer to directly metastasize to the mediastinum [5]. We present a rare case with the solitary pulmonary hilar lymph node. The thoracic surgeon should be aware of this rare presentation of otherwise, early colorectal cancer. We also review the impact of mediastinal lymphadenectomy on survival in colorectal cancers in the literature.

## 2. Case Report

A 75-year-old white lady was referred to the local cardiothoracic service for a new right hilar lymph node. She had two previous cancers treated (colon and breast). An extended right hemicolectomy and partial gastrectomy were performed in 2003 for a Dukes' stage B (T4, N0, M0) adenocarcinoma of the transverse colon. She then received adjuvant chemotherapy (5FU and folinic acid). Follow-up as per national guidelines included repeat CT and colonoscopies, confirming resection of cancer. In 2010, she was found to have primary right breast cancer which was treated by wide local excision and tamoxifen for 5 years.

**Figure 1:**
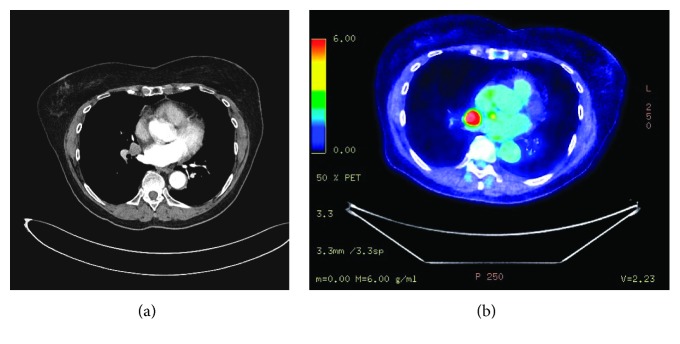
(a) CT scan and (b) PET scan showing PET-positive R12 node.

A screening colonoscopy identified a low sigmoid/upper rectal tumour in late 2015, with histology indicating a mucinous lesion with signet ring pathology. This was thought to be a new primary tumour as it was in a separate site (the upper rectum as opposed to the transverse colon), and it was 12 years after the first tumour. A 4 cm long stricture was also noted. CT and MRI were performed and showed a peritumour lymph node measuring 6 mm, potentially representing local spread, and a 15 mm lymph node in the right lung was also identified (T3N1M1). The right pulmonary lymph node was not present on a CT scan from 2010. PET scan (Figure 1) showed a PET avid R12 (SUV 12.8) node in the thorax, and it was confirmed on EBUS to be an adenocarcinoma of colorectal origin, while the peritumour abdominal lymph node was PET negative. Hence, it was decided to provide the patient with neoadjuvant chemoradiotherapy and then restage with a CT scan.

The patient finished her chemoradiotherapy (45 Gy and capecitabine), and the restaging scans showed complete response of the primary tumour in the rectum. It was decided to resect the pulmonary nodule as it was resistant to chemotherapy.

Surgical excision of the right hilar lymph node was performed by a posterolateral thoracotomy. During the procedure, a moderate size (3 × 3 cm) lesion in the right hilum was isolated. Histology review reported a moderately differentiated adenocarcinoma, keeping in appearance with metastatic colorectal adenocarcinoma.

Following surgery, the patient developed left lower lobe (contralateral) collapse requiring prolonged stay in the intensive treatment unit. On resolution of the lung collapse, she was referred back to her local hospital.

Further restaging scans showed evidence of a new 3 mm right pulmonary nodule and extensive hilar lymph node enlargement. Revisiting the chest for surgical clearance was not possible due to the fitness of the patient and the stormy postoperative period following the initial surgery. The patient was also not fit for chest irradiation.

She was thereafter put on the palliative pathway. Her thoracic disease remained stationary during the follow-up period.

## 3. Discussion

The lungs remain a common metastatic target for a variety of cancers. It is not uncommon for pulmonary metastases to further spread to the lymphatic drainage of the area. It is however extremely rare for isolated lymph node metastases to present in patients with metastatic colorectal cancer without any other organ involvement [6]. Lymph node involvement in colorectal cancer appears to have an ominous prognosis [7]. In our case, the route of metastasis is of particular importance, and the team has put forward a number of hypotheses. Firstly, the solitary pulmonary lymph node could present a catchment area for an early small pulmonary metastasis. The CT-PET scan picked up the lymph node, while the original pulmonary metastasis was too small. The next hypothesis was that the metastasis to the right pulmonary node was via the para-aortic lymphatic system, originating from small peritumour lymphatics associated with the primary adenocarcinoma. Finally, the tumour could have spread by the paravertebral venous plexus, bypassing both the liver and the para-aortic lymph nodes, to reach the pulmonary lymph nodes [5].

The management of such rare cases remains a puzzling conundrum for modern clinicians, as no guidelines exist and evidence arises only from case series. Experience from previous studies supports excision of pulmonary metastasis alongside lymph node excision [4] in selected patients, with excision of solitary nodules reporting favourable prognosis [8]. Villeneuve and Sundaresan proposed an evidence-based algorithm with regard to which patients are suitable for such cases and what the most appropriate intervention is, depending on presentation [9]. Parameters to consider include resectability, isolation of disease, patients' fitness level, presence of bilateral/unilateral disease, size, and location.

However, this algorithm involves pulmonary metastasis and does not focus on intrathoracic lymphadenopathy. Management of metastatic lymphadenopathy in the chest, especially mediastinal, could involve surgery, radiotherapy, or chemotherapy. The surgical and radiotherapy options are usually a part of the management of the original pulmonary metastasis. For this reason, data are lacking on how to locally treat intrathoracic lymph node metastasis with no pulmonary disease. In our case, the response to chemotherapy was complete with the intra-abdominal disease, but the thoracic disease was resistant. This could be due to the fact that the metastatic disease was secondary to a more aggressive subgroup of cells that have further mutations allowing them to detach, migrate, and “arrest” in a distant environment [10].

## 4. Conclusion

The ominous prognosis of mediastinal lymph node metastasis and the improved prognosis following a more aggressive mediastinal clearance while performing pulmonary metastasectomy, for colorectal metastasis in particular, suggest that performing a mediastinal clearance may be justifiable [3, 5], if not favourable. Further studies and meta-analyses are needed to solidify such a conclusion.
